# Airway macrophage specific glycocalyx expression and remodeling following viral infection

**DOI:** 10.1016/j.matbio.2026.102033

**Published:** 2026-06-27

**Authors:** Ziyun Zhang, Iashia Z Nabi, Samie Dalgarno, H. Davies-Strickleton, Ilayda Akbulut, Tracy Hussell, Douglas P Dyer

**Affiliations:** a Lydia Becker Institute of Immunology and Inflammation, Faculty of Biology, Medicine and Health, Manchester Academic Health Science Centre, University of Manchester, United Kingdom; b Manchester Cell-Matrix Centre, Faculty of Biology, Medicine and Health, Manchester Academic Health Science Centre, University of Manchester, United Kingdom; c Geoffrey Jefferson Brain Research Centre, Manchester Academic Health Science Centre, Northern Care Alliance NHS Group, University of Manchester, Manchester, United Kingdom

**Keywords:** Macrophage, Glycocalyx, Virus, Infection

## Abstract

The cell surface glycocalyx is a pericellular matrix that surrounds all cells, including macrophages, and regulates their function and interaction with the environment. The glycocalyx is composed of a range of glycans and glyco-proteins and its shedding into the blood is observed in, and is used as a marker of, a variety of inflammatory conditions such as viral infections. However, little is known about the signals and locations that regulate glycocalyx formation and heterogeneity across macrophage subsets. We now report that mouse lung macrophages express a glycocalyx that is dependent on their anatomical location and the mediators driving their differentiation from monocytes. Furthermore, during inflammatory conditions, caused by viral infection, the macrophage glycocalyx is remodelled in complex ways. Overall, our study provides a novel pathway involved in macrophage glycocalyx formation at rest and during inflammatory responses at mucosal tissue sties. This impacts our emerging understanding of the glycocalyx on immune cells beyond the classical paradigm of glycocalyx regulation of endothelial and vascular function.

## Introduction

The glycocalyx is a meshwork of different glyco-conjugates that forms a pericellular matrix on the surface of most cells [1]. Its function ranges from barrier formation to transduction of signals from the extracellular environment [2]. Proteoglycans are key components of the glycocalyx and are composed of protein cores with sulfated glycosaminoglycan (GAG) sugar side chains (*e.g*. heparan sulfate (HS) and chondroitin sulfate (CS)). The glycocalyx also contains the unsulfated GAG hyaluronan (HA) [3]. Proteoglycans that tether the glycocalyx to the cell surface include syndecans (SDC) 1–4. These transmembrane tethers provide a means for the cell to sense their local microenvironment via the glycocalyx in addition to their role as receptors for chemokines [4–7].

This glycocalyx meshwork is most studied on endothelial cells where it regulates cell extravasation through steric hindrance of homing receptors and/or by electrostatic repulsion and vascular homeostasis [8]. The glycocalyx on endothelial cells and airway epithelial cells is shed on injury [9], and in response to LPS [10], influenza virus [11] and bleomycin [12], leading to barrier dysfunction, epithelial apoptosis, surfactant dysfunction, impaired epithelial repair and susceptibility to secondary bacterial pneumonia [11]. The method of glycocalyx shedding seems to depend on location. Small fragments, like HS, are released into the blood by heparanase, whereas larger fragments in the airway indicate shedding of the tethering proteoglycans, such as SDC-1 [13,14].

More recently, a glycocalyx has been described on other cell types, *e.g*. monocytes, where it regulates their migration into tissues, and also on macorphages [15–17]. The macrophage glycocalyx is key to their functionality [18], including regulation of phagocytosis [15], chemokine signaling [19], motility [20] and disease pathogenesis [21,22].

The heterogeneity of glycocalyx on cells in different anatomical locations is largely untested, but has recently been explored in the endothelial compartment across tissues [23,24]. The regulation of the macrophage glycocalyx across anatomical locations is particularly important because limitation of macrophage reactivity in the airspaces is important to prevent overt inflammation to innocuous antigens or commensal microbes [25]. Numerous site-specific mechanisms exist that raise the threshold for airway macrophage activation. These include ligation of airway macrophage CD200 receptor by epithelial expressed CD200 [26], lung epithelial-derived surfactant proteins binding and blocking macrophage toll-like receptors [27] and enhanced concentrations of airway IL-10, Granulocyte-macrophage colony-stimulating factor (GM-CSF), activated TGF-beta and many more [28]. However, little is known about whether the macrophage glycocalyx varies across, and is regulated by, tissue location and associated cytokine signalling. Therefore, we hypothesised that the lung macrophage glycocalyx is context-specific and regulated by the local tissue niche environment.

We now show that a distinct glycocalyx layer surrounds airway, but not lung interstitial, macrophages at steady state, which we reproduce *in vitro* by differentiating monocytes into macrophages with GM-CSF (but not Macrophage colony-stimulating factor (M-CSF)), followed by further polarisation with IL-4. Interestingly, interstitial macrophages migrating into the lung during inflammation gain a dense glycocalyx, as do transferred monocyte-derived macrophages. This demonstrates that the airway microenvironment facilitates the acquisition of a glycocalyx on macrophages, which potentially regulates their function.

## Results

**Fig. 1:**
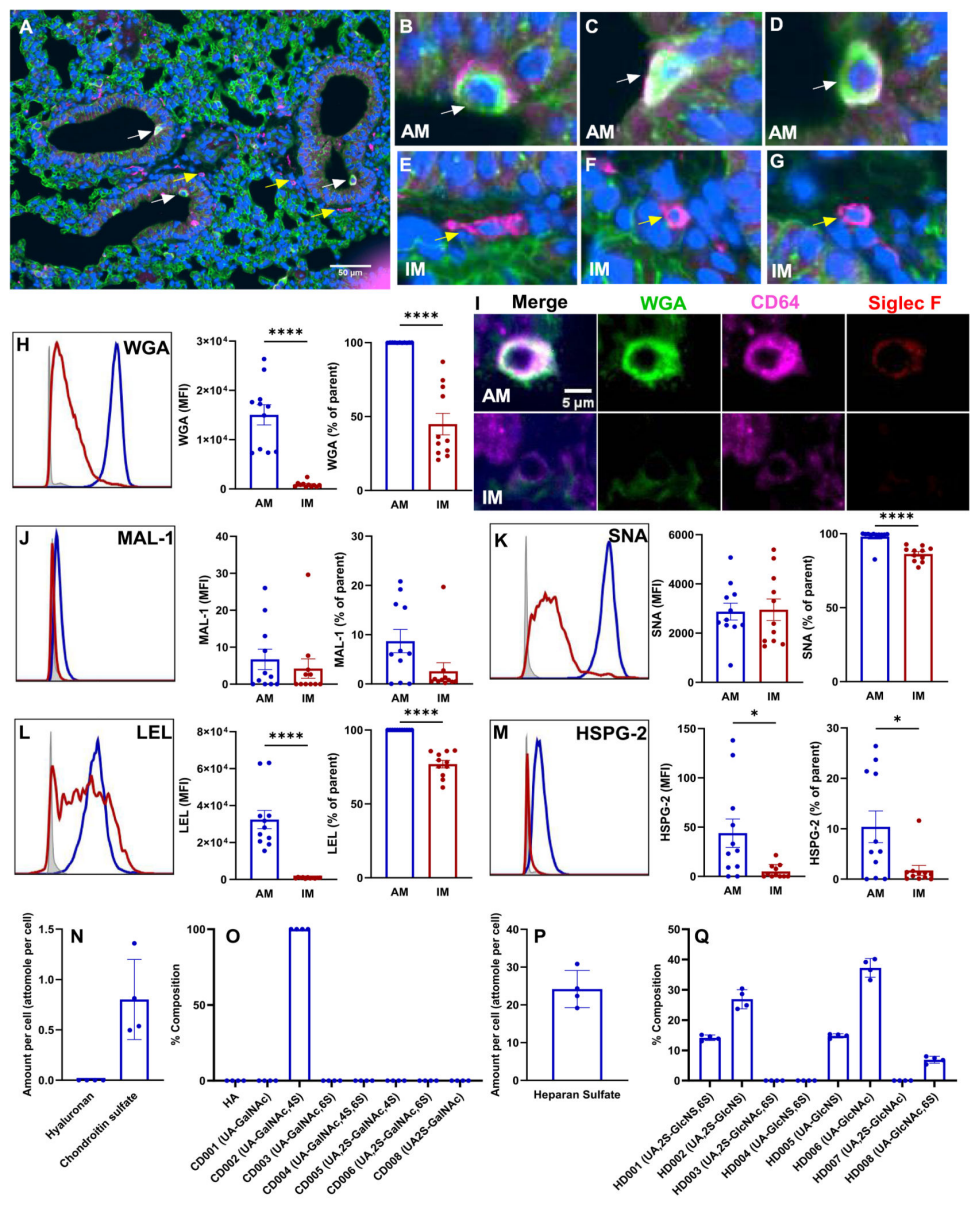
Macrophages in the lung and airway express the glycocalyx. Representative Confocal images of a healthy mouse lung showing general glycocalyx (WGA lectin: green), macrophages (CD64: Magenta), and DNA material (DAPI: Blue). Scale bar: 50 μm. Macrophages are located in the airway (white arrows) and in the tissue (yellow arrows) (*n* = 3) (**A**). Detailed views of airway macrophages (**B, C, and D**). Detailed views of tissue macrophages (**E, F, and G**). Cells were isolated from the digested lungs of female C57BL/6 mice to identify alveolar macrophages, and interstitial macrophages. Histogram comparison and quantification of fluorescence signals from WGA lectin from alveolar macrophages (blue), interstitial macrophages (red), and fluorescence minus one control (FMO) (grey) (*n* = 11) (**H**). Representative confocal images of healthy naïve mouse lungs showing general glycocalyx (WGA lectin: green), macrophage marker (CD64: magenta), alveolar macrophage marker (Siglec-F: red) and DAPI (blue) (*n* = 7) (**I**). Histogram comparison and quantification of fluorescent signals from lectins MAL-1 (J), SNA (K) and LEL (L) or HSPG-2 antibody (M) binding to alveolar macrophages (blue), interstitial macrophages (red), and fluorescence minus one control (FMO) (grey) (*n* = 11 for **J-M**). BAL cells (alveolar macrophages) were analysed by HILIC-MS/MS for (**N**) total CS and HA disaccharide content, (**O**) CS disaccharide composition, (**P**) total HS disaccharide content and (**Q**) HS disaccharide composition. Each dot represents an individual mouse with data pooled from three independent experiments (*n* = 4 for **N-Q**). Data are plotted as the mean ± SEM and analysed using unpaired *t*-tests. *, *P* ≤ 0.05; **, *P* ≤ 0.01; ***, *P* ≤ 0.001; ****, *P* ≤ 0.0001.

The relative abundance of glycocalyx on lung macrophages occupying different anatomical compartments is unknown. Fluorescently labelled Wheat Germ Agglutinin (WGA) that binds glycoconjugates (N-acetylglucosamine and sialic acid residues) on cell membranes, was used to visualise the macrophage glycocalyx in lung paraffin-embedded tissue sections [29,30]. We observed a glycocalyx on macrophages in the airway lumen adjacent to the airway epithelium (Fig. 1A-D). By contrast, CD64+ macrophages in the lung interstitial tissue showed little binding to WGA (Fig. 1A, E-G). A similar result was also obtained after retrieving airway macrophages and interstitial macrophages by tissue digestion and analysing glycocalyx expression by flow cytometry (Fig. 1H) distinguished based on the staining protocol shown in supplementary Fig. 1. Macrophages expressing WGA-binding glycans in the airways (but not the interstitial macrophages) were confirmed in a separate experiment as double positive for CD64 and Siglec F (Fig. 1I) [31].

In order to determine which glycans may be present within the glycocalyx on the airway macrophages we firstly performed flow cytometry analysis using a panel of lectins (Fig. 1J-L) that bind to a range of different glycans. This analysis revealed comparable binding of *Maackia amurensis* lectin (MAL-1, which preferentially binds to terminal 3-O sulfated Gal on LacNAc [32]) between alveolar and interstitial macrophages. We also observed higher levels of binding of Sambucus Nigra Lectin (SNA, which binds preferentially to sialic acid on a terminal galactose [32]) and Lycopersicon Esculentum lectin (LEL, which binds to GlcNAc oligomers [32]) to alveolar compared to interstitial macrophages. These data further detail differences in the glycans contained within the glycocalyx of alveolar and interstitial macrophages.

Secondly, we determined that the proteoglycan HSPG-2 was present on alveolar macrophages but not interstitial macrophages (Fig. 1M). As a result, we then utilised disaccharide analysis to determine the presence and composition of GAGs on the alveolar macrophage (from BAL) surface. With this approach we could detect CS, but not HA, on BAL cells (alveolar macrophages) (Fig. 1N). The dominant CS disaccharide was UA-GalNAc, 4S (Fig. 1O). These cells also contained heparan sulfate (Fig. 1P), with the main disaccharide being UA-GlcNAc (Fig. 1Q). Interestingly, the HS contained relatively high proportions of UA,2S-GlcNS,6S (approximately 14%) and UA,2S-GlcNS (approximately 30%), compared to other sources of biologically derived HS [33,34]. Together these data demonstrate the presence of a tissue location specific macrophage glycocalyx containing a range of different N-linked glycans and proteoglycans.

**Fig. 2:**
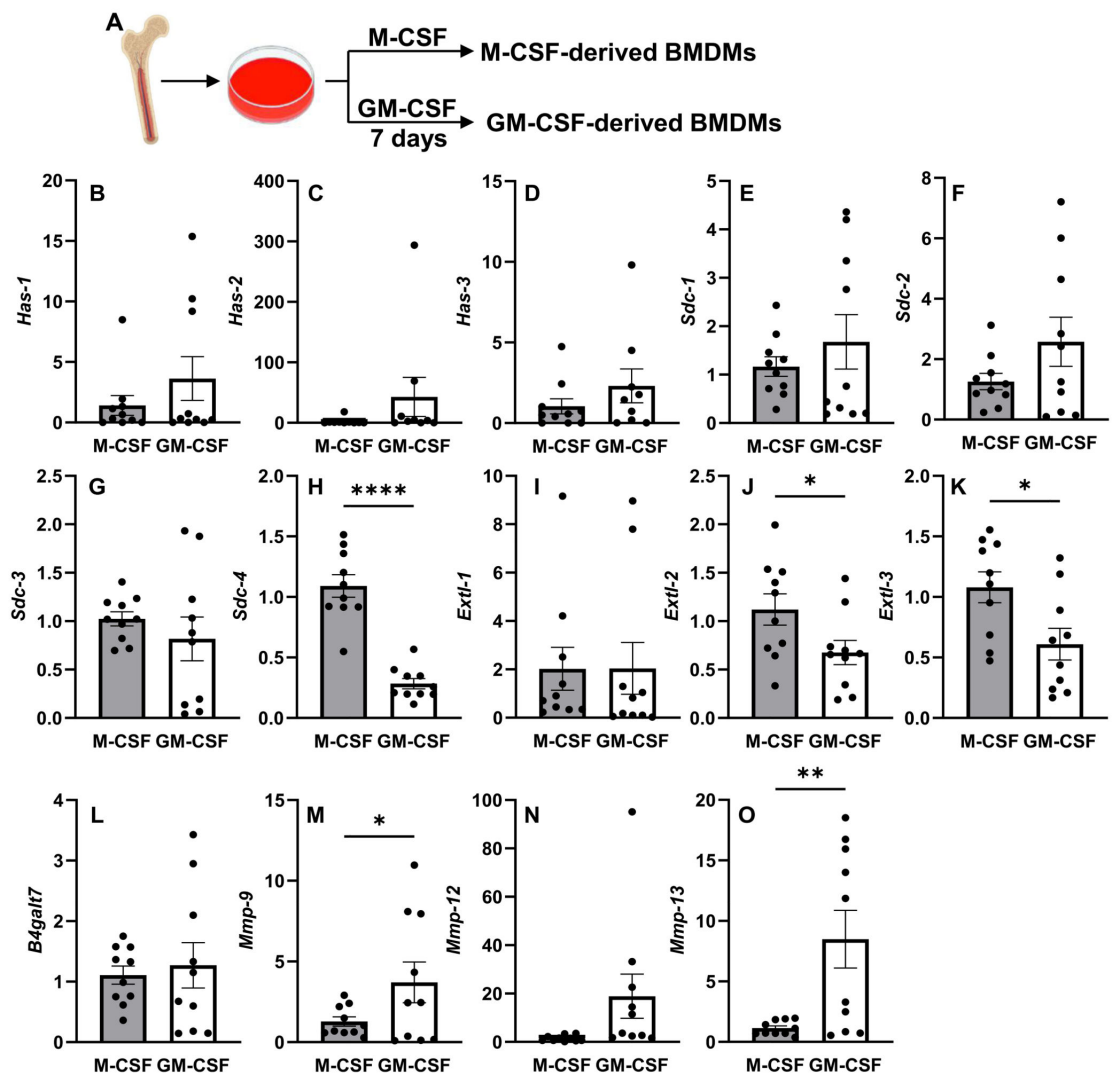
GM-CSF and M-CSF-derived BMDMs express differential levels of genes that regulate glycocalyx synthesis and degradation. Bone marrow cells were derived with M-CSF or GM-CSF (A). The glycocalyx-relative mRNA expressions were measured in GM-CSF- and M-CSF-differentiated BMDMs. RT-qPCR data are normalised to the housekeeping genes *b2m* and displayed as fold change over the M-CSF-derived BMDMs. *Has-1* (B), *has-2* (C), *has-3* (D), *sdc-1* (E), *sdc-2* (F), *sdc-3* (G), *sdc-4* (H), *extl-1* (I), *extl-2* (J), *extl-3* (K), *b4galt7* (L), *mmp-9* (M), *mmp-12* (N), and *mmp-13* (O). Each dot represents a biological repeat (cells derived from a different individual mouse), data are from three independent experiments pooled (*n* = 10), plotted as the mean ± SEM and analysed using unpaired *t*-tests. *, *P* ≤ 0.05; **, *P* ≤ 0.01; ***, *P* ≤ 0.001.

To understand what factors might drive glycocalyx expression on airway macrophages (and whether they produce it themselves), we differentiated bone marrow mesenchymal stem cells into macrophages (BMDMs) using GM-CSF or M-CSF (Fig. 2A) [35]. Importantly, GM-CSF is highly expressed in the airspaces and can therefore be used to model the effect of this location on macrophage differentiation [36]. We firstly confirmed that the progenitor cells showed evidence of a minimal WGA detectable glycocalyx (Supplementary Fig.. 2 A). Both GM-CSF and M-CSF-derived BMDMs bound highly to WGA and expressed SDC-1 and CD44 by flow cytometry analysis (supplementary Fig. 2). We then analysed the transcriptional levels of a range of genes that regulate the synthesis of glycocalyx GAGs, protein cores or degradation enzymes [24]. RT-qPCR revealed that RNA levels for some genes involved in glycocalyx synthesis and regulation were significantly lower in GM-CSF-derived, compared to M-CSF-derived BMDMs. This included lowered mRNA for *sdc-4, extl-2, and extl-3* (Fig. 2B-L). We also assessed levels of MMP genes, as these enzymes can degrade and remodel the glycocalyx. *Mmp-9* and *mmp-13* were significantly raised in GM-CSF-derived BMDMs, presented as a fold change of GM-CSF/M-CSF BMDMs (Fig. 2 M-O) or as relative expression levels over the housekeeping gene *b2m* (Supplementary Fig. 3). Together these data demonstrate differential effects of M-CSF and GM-CSF on macrophage expression of the genes that regulate glycocalyx synthesis and degradation. Demonstrating the potential of tissue location specific signals to regulate the cell surface glycocalyx on macrophages.

**Fig. 3:**
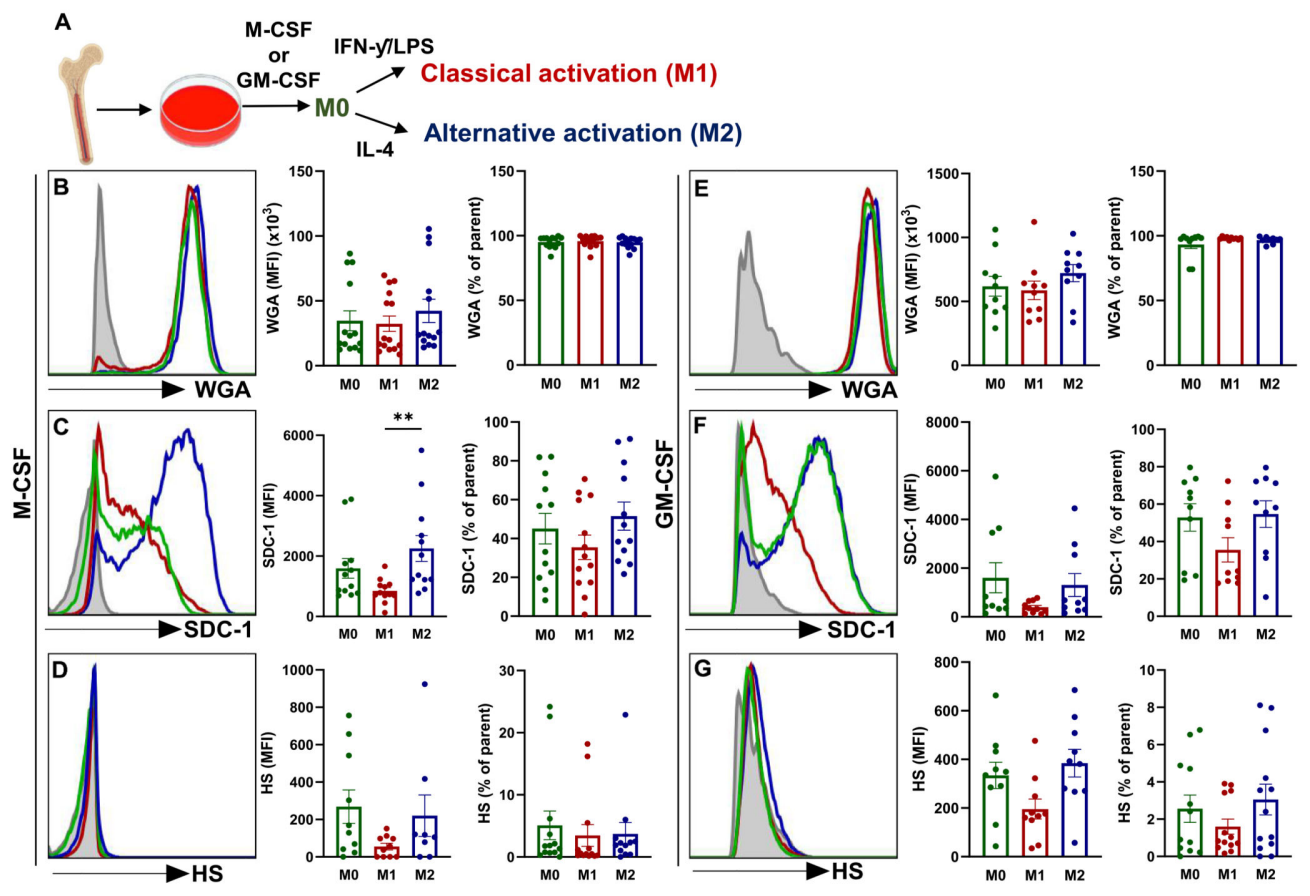
Classical or alternative activation of M-CSF and GM-CSF-derived BMDMs regulates their glycocalyx content. The femurs of C57BL/6 female mice were flushed with HBSS to collect bone marrow cells. The bone marrow cells were then cultured with 20 ng/ml M-CSF, or GM-CSF for 7 days. After 7 days, the cells were left unstimulated (M0) (green), polarised to classically (M1) (red) or alternatively activated (M2) (blue) macrophages (**A**). The representative histograms, MFI and percentage of M-CSF-derived BMDMs positive for glycocalyx as indicated by WGA lectin (**B**), SDC-1 (**C**), and HS (**D**). The MFI and percentage of GM-CSF-derived BMDMs positive for glycocalyx as indicated by WGA lectin (**E**), SDC-1 (**F**), and HS (**G**). Each dot represents a biological repeat (cells derived from a different individual mouse), M-CSF and GM-CSF data are each pooled from four independent experiments (*n* = 13 in M-CSF group, *n* = 10 in GM-CSF group), plotted as the mean ± SEM and analysed using an ordinary one-way ANOVA and Tukey’s multiple comparisons tests. *, *P* ≤ 0.05; **, *P* ≤ 0.01.

The effect of macrophage polarisation on their function has been a key research focus for a number of years, to understand their wide-ranging role in the immune response [37]. Therefore, we hypothesised that macrophage polarisation would also influence their cell surface glycocalyx, given its’ importance to their function. To test this hypothesis, we determined whether further polarisation of M-CSF-derived BMDMs with IFN-ƴ/LPS or IL-4 (Fig. 3A) affected glycocalyx expression [35]. The effectiveness of polarisation was determined by the relative mRNA expression of *nos-2* and *chi3l-1*, which, as expected, were raised in M1 and M2 polarising stimuli [38], respectively (Supplementary Fig. 4). No difference in the already high levels of WGA binding was observed between the different conditions (Fig. 3B). In M-CSF-derived macrophages, SDC-1 was low in non-polarised macrophages and those polarised with IFN-ƴ/LPS, but significantly greater expression occurred in the presence of IL-4 (Fig. 3C). No difference was observed in the low levels of HS when analysed by flow cytometry (Fig. 3D). For GM-CSF-derived BMDMs, no significant difference was observed for WGA, SDC-1 or HS levels (Fig. 3E-G). Together these data suggest that additional polarisation with type 1 (IFN-γ/LPS) or type 2 (IL-4) cytokines, in these conditions, had minimal additional effects on the presence/content of the macrophage glycocalyx.

**Fig. 4:**
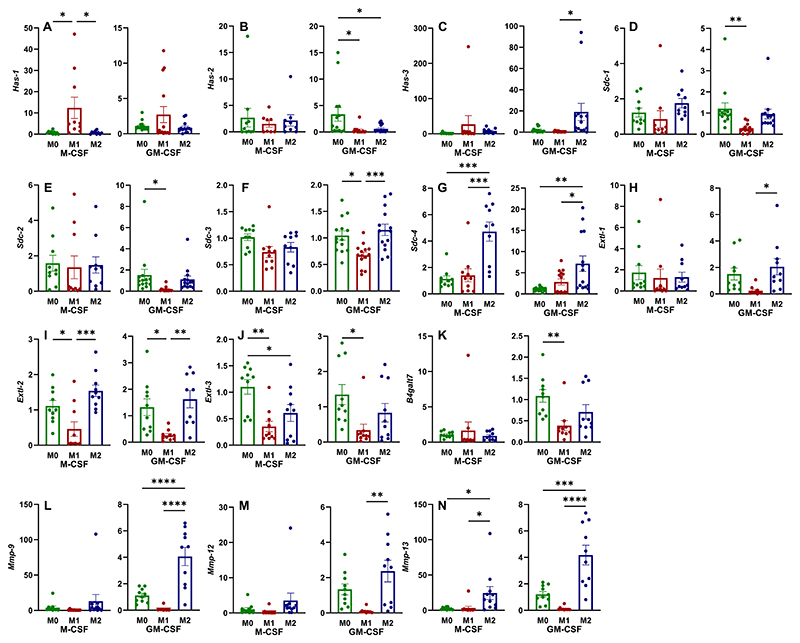
Classical activation of BMDMs reduced the transcription of most glycocalyx-related genes. M-CSF-derived BMDMs and GM-CSF-derived BMDMs were left unstimulated (green) or polarised to classically (M1) (red) and alternatively activated (M2) (blue) macrophages. The relative mRNA expressions were measured and displayed as fold change over the level of unpolarised BMDMs (set as 1). *Has-1* (**A**), *has-2* (**B**), *has-3* (**C**), *sdc-1* (**D**), *sdc-2* (**E**), *sdc-3* (**F**), *sdc-4* (**G**), *extl-1* (**H**), *extl-2* (**I**), *extl-3* (**J**), *b4galt7* (**K**), *mmp-9* (**L**), *mmp-12* (**M**), and *mmp-13* (**N**). Each dot represents a biological repeat (cells derived from a different individual mouse), data are from three independent experiments pooled (*n* = 10 in M-CSF group, *n* = 14 in GM-CSF group), plotted as the mean ± SEM and were analysed using an ordinary one-way ANOVA and Tukey’s multiple comparisons tests. *, *P* ≤ 0.05; **, *P* ≤ 0.01; ***, *P* ≤ 0.001; ****, *P* ≤ 0.0001.

To assess the effect of polarisation on glycocalyx production at the transcriptional level we analysed the mRNA of lysed M-CSF- and GM-CSF-derived BMDMs, with IFN-ƴ/LPS or IL-4 polarisation (Fig. 4). We observed that the expression of glycocalyx content synthesis genes altered with different stimuli. For example for the HA synthesis genes, IFN-ƴ/LPS induced *has-1* transcription (Fig. 4A) but reduced *has-2* transcription (Fig. 4B) and IL-4 induced *has-3* transcription (Fig. 4C). Furthermore for the proteoglycan core genes, *sdc-1*, −*2 and* −*3* expression was reduced in response to IFN-ƴ/LPS (Fig. 4D-F), while the expression of *sdc-4* was enhanced by IL-4 (Fig. 4G). Finally IFN-ƴ/LPS also caused reductions of *extls* and *b4galt7* that drive the biosynthesis of HS (Fig. 4H-K) [39]. Therefore, stimuli driving type 1 cytokines inhibited the majority of assessed glycocalyx synthesis-related gene expression in macrophages.

In contrast *Mmp-9*, −*12*, and −*13* were induced by the IL-4 polarisation (Fig. 4l-N). Despite the fold changes, the relative amount of transcription of *has, sdc* and *extl* families of genes were low in comparison to the amount of the housekeeping gene *b2m* (Supplementary Fig. 5). These data suggest that IL-4 stimulation may promote glycocalyx and/or ECM remodelling via production of matrix degrading enzymes by macrophages.

**Fig. 5:**
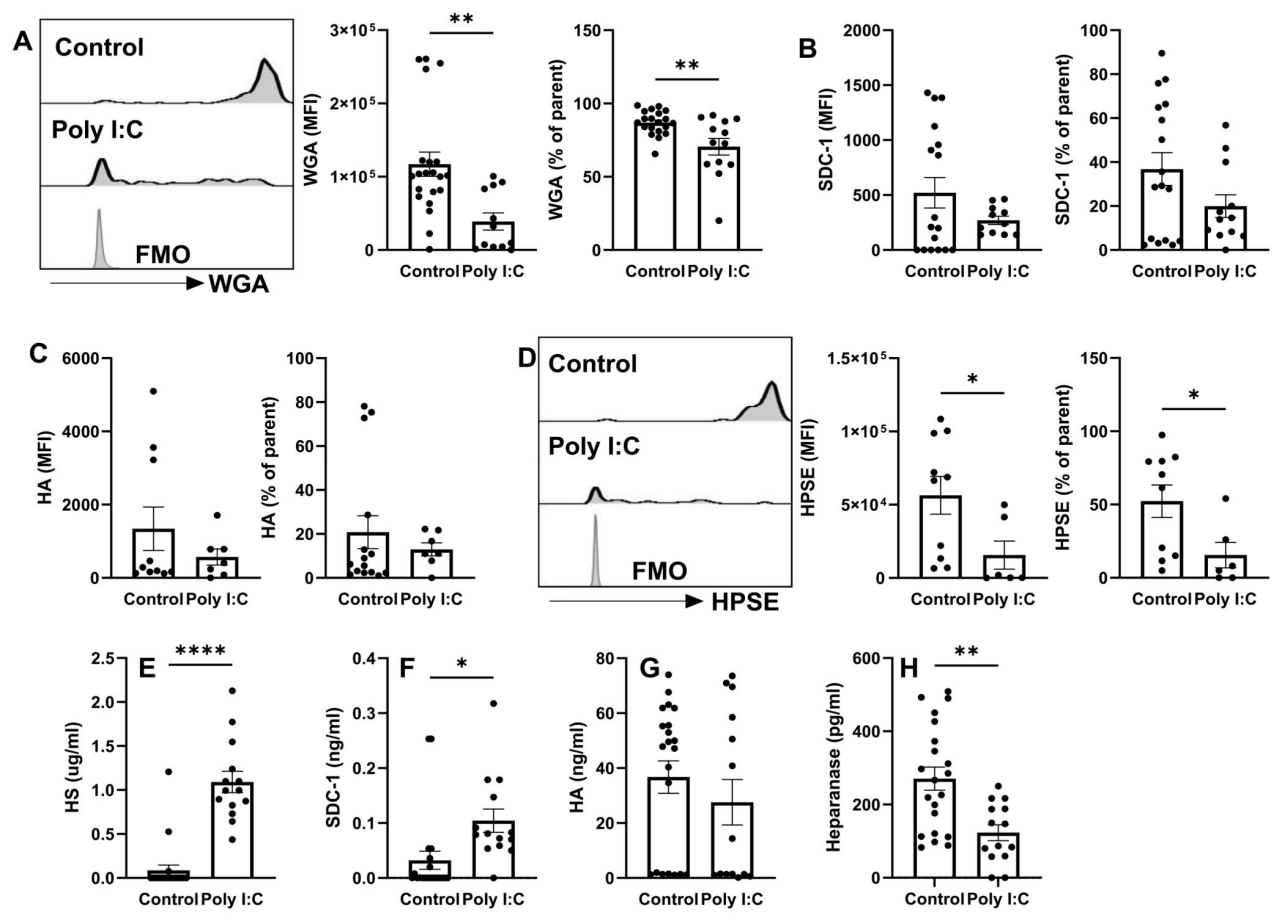
Incubation of mouse BAL fluid AMs with Poly I:C reduces the glycocalyx on the cell surface. AMs collected from mouse BAL fluid were incubated with 10 µg/ml Poly I:C for 24 h or tissue culture medium alone. Representative histogram of the lectin WGA staining: 10 µg/ml of Poly I:C treatment, medium-only control, and FMO control. MFI and percentage of positive cells of WGA lectin staining (**A**). Cell surface levels of glycocalyx components are shown by SDC-1 (**B**), and HA (**C**). Representative histogram of intracellular heparanase: 10 µg/ml of Poly I:C treatment, medium-only control, and FMO control. MFI and percentage of positive cells of intracellular heparanase (**D**). The cell culture supernatant was collected after 24 h of stimulation and tested by ELISA for HS (**E**), SDC-1 (**F**), HA (**G**), and heparanase (**H**). Each dot represents a biological repeat, data are from five independent experiments pooled (*n* = 20), plotted as the mean ± SEM and were analysed using an ordinary one-way ANOVA and Tukey’s multiple comparisons tests, or unpaired *t*-tests. *, *P* ≤ 0.05; **, *P* ≤ 0.01; ***, *P* ≤ 0.001; ****, *P* ≤ 0.0001.

Given the reduction of the macrophage glycocalyx synthesis gene expression by type 1 cytokines stimuli we next assessed the effect of Poly I:C (a viral mimetic that induces a type-1 inflammatory response [40]) on alveolar macrophages collected from mice bronchoalveolar lavage (BAL) fluid. Incubation with Poly:IC reduced WGA detectable glycocalyx (Fig. 5A) but did not affect SDC-1 and HA on the cell surface (Fig. 5B-C). The Poly I:C treatment also reduced the intracellular level of heparanase (Fig. 5D), suggesting it may have been released by the cell in response to the stimulus. In the cell culture supernatant, we observed a greater presence of soluble HS and SDC-1 that has been released from Poly I:C treated AMs (Fig. 5E, F), but not HA (Fig. 5G). The Poly I:C treatment also reduced the heparanase level in the cell culture supernatant (Fig. 5H). Together these data confirm that response to mimics of viral infection induces remodelling of the alveolar macrophage glycocalyx in culture.

**Fig. 6:**
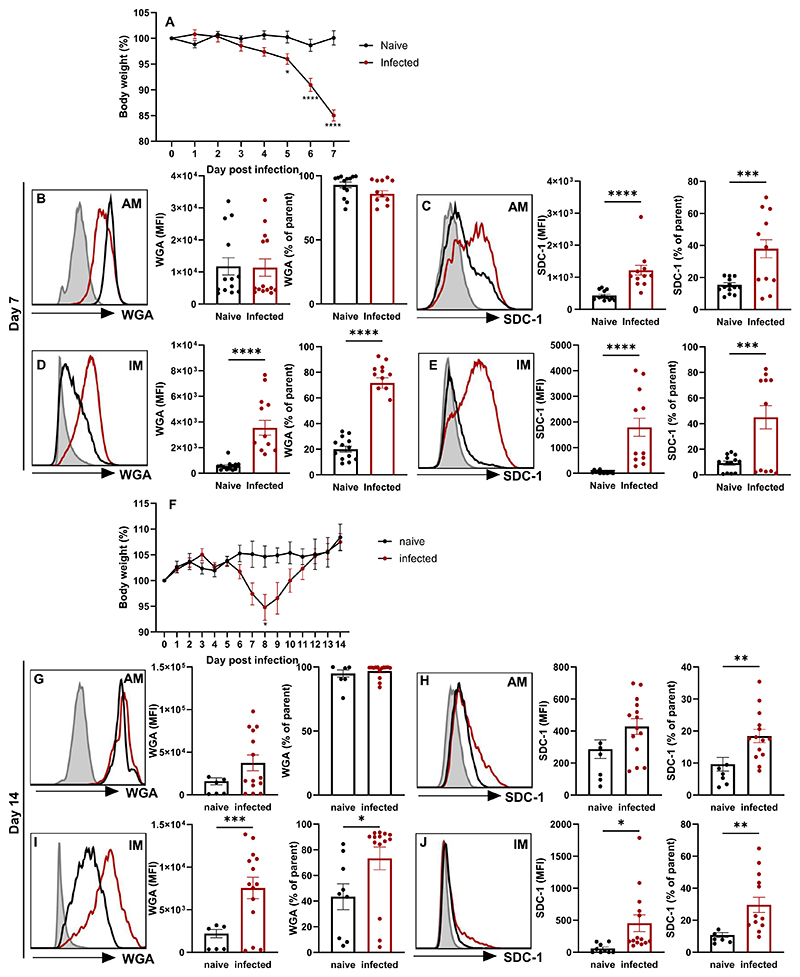
Influenza virus infection promotes SDC-1 expression on both alveolar macrophages and interstitial macrophages. At day 0, mice were intranasally given 5 PFU IAV PR8 or PBS in the naïve control group. On day 7, the lung from both groups was harvested. The lung was digested and stained for flow cytometry analysis. Average weight change of naïve and infected mice from day 0 to day 7 (**A**). The MFI, and percentage, of general glycocalyx were shown by WGA lectin staining of AMs (**B**) and IMs (**D**) on day 7. The median fluorescence intensity (MFI), and percentage, of SDC-1 of AMs (**C**) and IMs (**E**) on day 7. The influenza virus infection experiment was repeated, and the mice were harvested on day 14. Average weight change of naïve and infected mice from day 0 to day 14 (**F**). The MFI and percentage of WGA lectin (**G**), and SDC-1 (**H**) of AMs on day 14. The MFI and percentage of WGA lectin (**I**), and SDC-1 (**J**) of IMs on day 14. Naive (black) and infected (red). Each dot represents an individual mouse, data are pooled from three independent experiments (Day 7 Naive, *n* = 14; Day 7 Infected, *n* = 14; Day 14 Naïve, *n* = 9; Day 14 Infected, *n* = 14), are presented as mean ± SEM and were analysed using unpaired *t*-tests. The significance of weight loss was analysed with two-way ANOVA. *, *P* ≤ 0.05; **, *P* ≤ 0.01; ***, *P* ≤ 0.001; ****, *P* ≤ 0.0001.

Since our *ex vivo* and *in vitro* studies suggest that the glycocalyx is reduced in the presence of type 1 cytokines, we next examined macrophages in an *in vivo* murine model of lung influenza virus infection. At day 7 after infection, the mouse weight loss is pronounced (Fig. 6A). Myeloid cells were gated as shown in Supplementary Fig. 6A. AMs maintained a high glycocalyx level, as shown by their WGA binding level irrespective of influenza virus infection (Fig. 6B), whereas they up-regulated SDC-1 (Fig. 6C). Interestingly, IMs up-regulated both the WGA staining (Fig. 6D), and SDC-1 after influenza virus infection (Fig. 6E). We also observed some differences in WGA, but not SDC-1, on other cell subtypes (Supplementary Fig. 6B-G). We next allowed the mice to recover from influenza virus infection and harvested tissues at day 14 post-infection (Fig. 6F). At this time point, we observed an up-regulation of SDC-1 but not glycocalyx (WGA binding) on alveolar macrophages (Fig. 6G, H). Again, IMs up-regulated both the WGA staining (Fig. 6I) and SDC-1 14 days after influenza virus infection (Fig. 6J). This suggests that, despite type 1 inflammatory cytokines reducing macrophage glycocalyx *in vitro*, the airway microenvironment *in vivo* overrides this and drives high macrophage glycocalyx levels even in the presence of viral triggers.

**Fig. 7:**
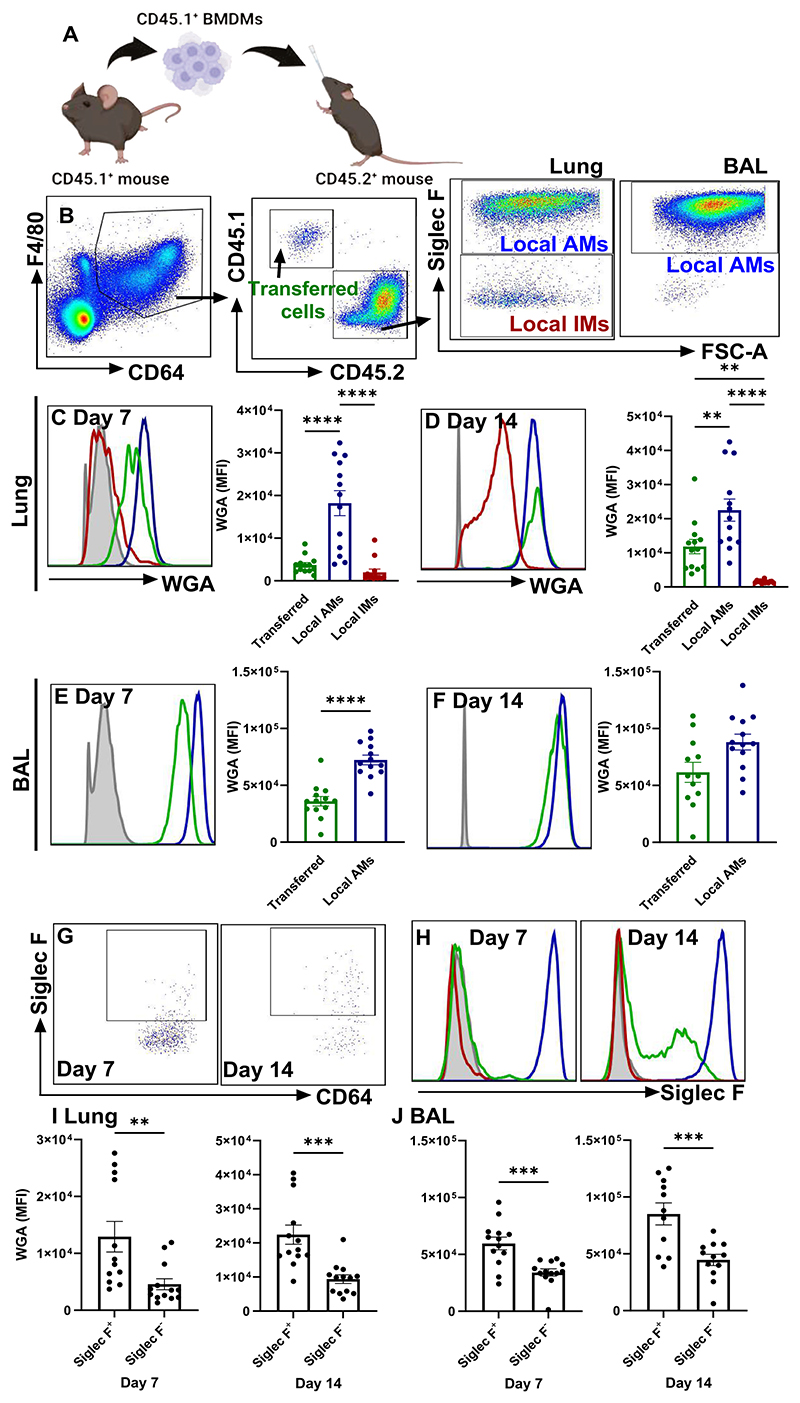
BMDMs transferred to the lung increased glycocalyx levels from day 7 to day 14. BMDMs pooled from 3 or 4 CD45.1^+^ mice were differentiated into macrophages with M-CSF and intranasally instilled in CD45.2^+^ wild-type C57BL/6 mice (**A**). At 7- or 14-day post-instillation, the BAL fluid and lungs from recipient mice were collected. The macrophages from collected cells were gated as CD64^+^ F4/80^+^, and further gated into transferred cells (CD64^+^ F4/80^+^ CD45.1^+^ CD45.2^-^); local AMs (CD64^+^ F4/80^+^ CD45.1^-^ CD45.2^+^ Siglec F^+^), and local IMs (CD64^+^ F4/80^+^ CD45.1^-^ CD45.2^+^ Siglec F^-^) (**B**). The WGA MFI of transferred cells, local AMs and local IMs collected from the digested lungs at day 7 (**C**) and day 14 (**D**). The WGA MFI of transferred cells and local AMs collected from the BAL fluid at day 7 (**E**) and day 14 (**F**). Representative flow plots of the transferred macrophages on the expression of Siglec-F on days 7 and 14 (**G**). Representative histograms of Siglec-F on days 7 and 14 (**H**). The transferred cells were divided into two subgroups based on the expression of Siglec-F. The MFI of WGA lectin staining from Siglec-F^+^ and Siglec-F^-^ transferred cells collected from the digested lung (**I**), and the BAL fluid (**J**). Each dot represents an individual mouse, data are pooled from three independent experiments (Day 7, *n* = 13; Day 14, *n* = 13) and are presented as mean ± SEM and were analysed using an ordinary one-way ANOVA and Tukey’s multiple comparisons tests, or unpaired *t*-tests. *, *P* ≤ 0.05; **, *P* ≤ 0.01; ***, *P* ≤ 0.001; ****, *P* ≤ 0.0001.

We next directly tested whether the airway microenvironment drives high levels of macrophage glycocalyx. To do so, we collected bone marrow from CD45.1^+^ CD45.2^−^ PeP-3 mice, differentiated them *in vitro* into macrophages with M-CSF (which are glycocalyx low) and transferred 0.5 × 10^6^ cells intranasally into CD45.1^-^ CD45.2^+^ C57BL/6 mice (Fig. 7A). BAL fluid and lung tissue were harvested 7 and 14 days later. The CD45.1^+^ transferred macrophages were clearly visible amongst the host CD45.2^+^ macrophages in the lung and airway. Local AMs and IMs were distinguished based on the expression of Siglec F (Fig. 7B). BAL does not remove the contents of the entire airways as the fluid doesn’t reach all of the alveolar sacs. Therefore, the digested lung always contains a proportion of residual airway macrophages, which are easily distinguished by flow cytometry. As expected, few Siglec F^-^ macrophages were isolated from the BAL, thus the transferred cells collected from the BAL fluid were only compared with Siglec F^+^ AMs (Fig. 7B). We compared the intensity from WGA among transferred CD45.1^+^, original local airway, and interstitial, macrophages from digested lung tissue. In the lung tissue at day 7, the greatest expression was observed on original local AMs that had not been removed by BAL (Fig. 7C). However, by day 14, both local and transferred macrophages in the lung expressed higher levels of WGA binding glycocalyx than the local IMs (Fig. 7D). This was also observed in cells recovered by BAL, where at day 7 there was still a lower level of glycocalyx on CD45.1^+^ transferred macrophages, compared to local AMs (Fig. 7E). By day 14 the transferred macrophages had accumulated comparable glycocalyx levels (WGA^+^) to the local AMs (Fig. 7F). Furthermore, as expected, transferred macrophages reaching the airway by day 7 of infection had lower Siglec F expression compared to original AMs. This also increased in proportion by day 14 (Fig. 7G, H), demonstrating development of the transferred cells into an AM-like phenotype. By focusing on the population of transferred macrophages that expressed higher levels of Siglec F we were able to demonstrate that these bound greater levels of WGA (indicative of glycocalyx) (Fig. 7I, J). Therefore, transferred cells enter the lung and airways and change their expression of glycocalyx, possibly via regulation of tethering receptors such as SDC-1 [18].

## Discussion

**Fig. 8:**
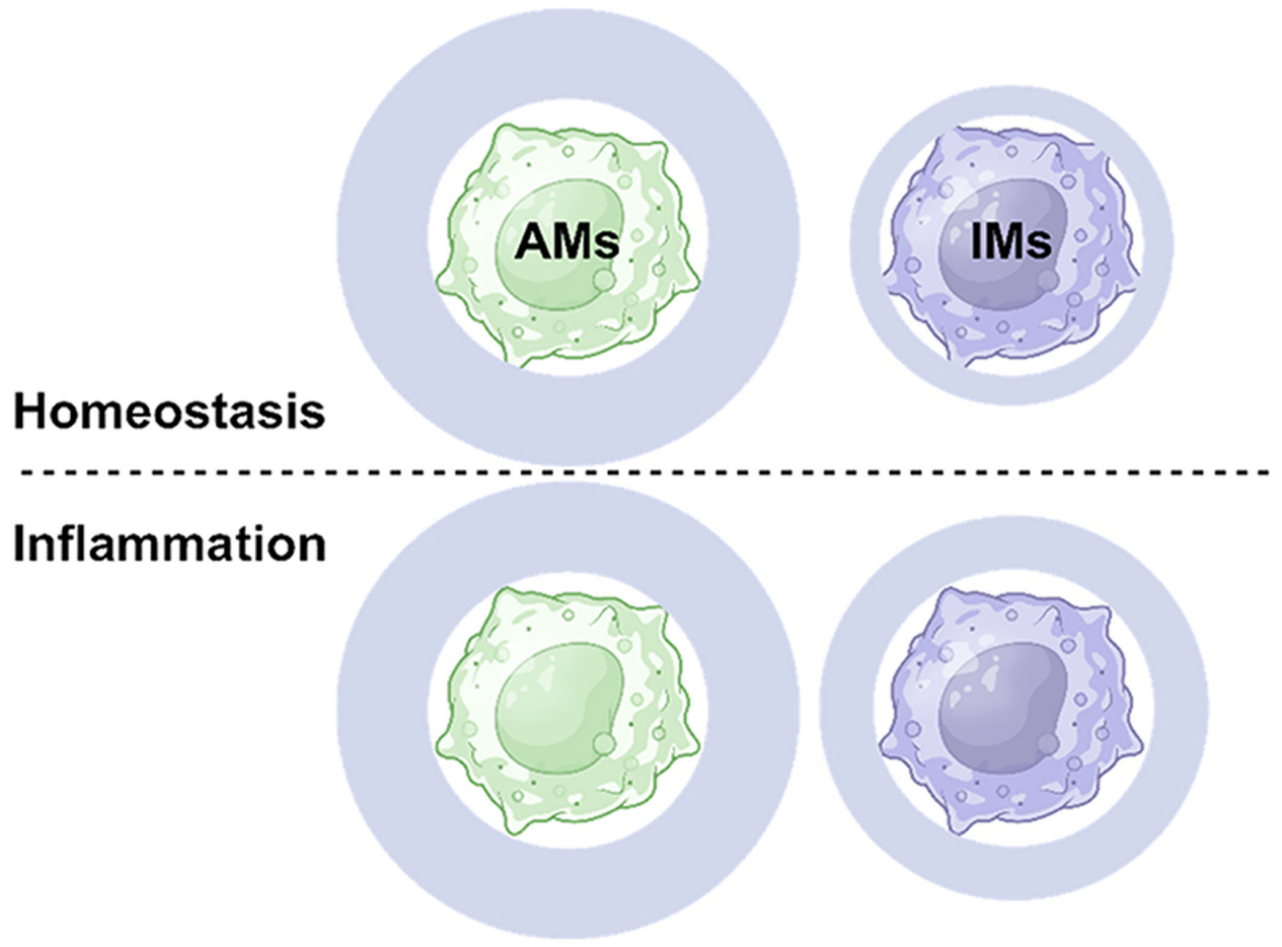
Airway macrophage specific glycocalyx expression and its remodelling following viral infection. Pulmonary macrophage glycocalyx is determined by anatomical location and inflammation. Airway macrophages (AMs) are decorated with high levels of glycocalyx in both homeostasis and inflammation. Interstitial macrophages (IMs) show low glycocalyx levels in homeostasis. During Influenza virus-induced inflammation, IMs increased the glycocalyx levels.

Here, we demonstrate that macrophages from distinct anatomical locations have a very different cell surface glycocalyx driven by local signals (*e.g*. GM-CSF) and inflammation. We used a number of different strategies to show that macrophage expression of the glycocalyx depends on where they reside in the lung (Fig. 8). First, confocal imaging reveals a glycocalyx on macrophages in the airspaces that is absent on those in the interstitium. Second, flow cytometry and disaccharide analysis demonstrated the specific glycan content of the alveolar macrophage glycocalyx. Third, bone marrow mesenchymal stem cells differentiated into macrophages with GM-CSF (highly expressed in the airspaces [36]) have differential RNA transcripts for glycocalyx synthesis proteins and their regulators. Further polarisation of GM-CSF monocyte-derived macrophages with IL-4 drives even greater expression of RNA and protein for glycocalyx components and regulators. Fourth, glycocalyx level increases on IMs at the later stages of an influenza infection, at a time when the virus is cleared and the repair process is beginning [41]. This could be driven by changes in existing IM populations or from alveolar macrophages infiltrating the interstitial space. It will also be important in future studies to determine the effect of alternative models (*e.g*. bacterial pneumonia). Fifth, glycocalyx-deficient macrophages instilled intranasally develop a glycocalyx in the airways over time.

There is support for a functional macrophage glycocalyx in the literature. For example, HS and CS attach to approximately 40 core proteoglycans, including perlecan, agrin, Collagen XVIII and SDCs, amongst others [42]. SDC-1 is expressed on macrophages in rheumatoid arthritis [21,43] and on human primary macrophages [19], and is likely to be decorated with HS and CS. Human macrophages polarised to an M2/regulatory phenotype also express higher SDC-1 [20]. Furthermore, cell-specific targeting of glycosaminoglycan chemical modification (sulfation) has been shown to affect macrophage function in other contexts [22,44]. Thus, evidence of macrophage SDC-1 and HSPG-2 expression provides the possibility for tethering of a glycocalyx, and the data corroborate the M2 bias of expression with the mRNA and protein we describe here.

The specific reduction of multiple glycocalyx components on M1 macrophages in our study may reflect reduced production or enhanced degradation. The effects we observe may also be specific to the experimental conditions used and could change under different stimulation conditions or timings. For example exotoses (EXT) 1 and 2 are involved in HS biosynthesis that occurs in the Golgi and the Golgi-endoplasmic reticulum interface [45,46]. Furthermore, biosynthesis of HS is blocked by knockdown of *ext1/2* and ext2/3-like (extl) genes [47]. Opposingly, TNF and IL-1 activate sheddases that cleave the glycocalyx [48], including from epithelial cells in acute respiratory distress syndrome [49] and also immune cells themselves [17], providing a source of biomarkers. Similarly neuraminidase 2 also causes glycocalyx shedding [50], and MMP9 liberates SDC-1 and – 4 from human macrophages [51]. Thus, the cell surface macrophage glycocalyx is a product of both synthesis and degradation pathways.

This raises the question of why it would be beneficial to reduce glycocalyx on macrophages, specifically in M1-dominated conditions. We hypothesise that sensing the glycocalyx through surface receptors triggers a functional programme of repair, and its absence may allow inflammation to proceed. This idea is supported by studies showing that attenuation of M1 macrophage polarisation and augmentation of M2 prevents glycocalyx shedding via a decrease in ADAM17 in a murine model of acute lung injury [52]. SDC-1 deletion, which, though not tested, would limit HS and CS binding, increases inflammation in murine asthma models [53,54]. Deficiency of EXTL2 (that drives HS production) promotes inflammation via TLR4 [55] and a variant of EXTL2 is associated with asthma exacerbation [56]. Again, the glycocalyx in these studies was not directly tested. Inflammation also accompanies a reduction of CS in mice [57]. Administration of CS reduces cytokines in fungal infection [58], CpG-induced IL-6 in a macrophage cell line [59] and peritoneal fibrosis via suppression of NF-κB [60], suggesting that reduced glycocalyx promotes inflammation. Limitation of sheddases (that would cleave glycocalyx tethers) also attenuates the inflammatory environment in sepsis [61]. The predominant focus of these studies is the glycocalyx on epithelium and endothelium. Our combined data, including *in vitro* polarisation and analysis of influenza virus recovery and repair, suggest that the dominant anti-inflammatory activity of the glycocalyx may be via modulation of macrophage inflammatory tone.

We used soluble TLR agonists for *in vitro* mechanistic studies, as a glycocalyx is reported to be necessary for endocytosis of viruses and bacteria and so *in vivo* models may have confounding issues. For example, proteoglycans, which rely on B4GALT7, Extl1 and Extl2 for the attachment of HS and CS, are implicated in the endocytosis of influenza virus [62,63]. Furthermore, CRISPR-Cas9 deletion of GAG biosynthesis regulators, including B4GALT7, reduced the uptake of *Mycobacterium absessus* in macrophages, possibly by modulation of integrin accessibility [64].

It is possible that modulation of inflammatory mediator production by macrophages following glycocalyx shedding may result from the unshielding of activating receptors [65]. However, the glycocalyx, when intact, may provide tonic signals to macrophages, limiting their inflammation, much in the same way that lung surfactant proteins reduce macrophage endocytosis and TLR signalling [27]. In general, receptor binding of individual glycocalyx components promotes an anti-inflammatory state in macrophages [54]. CS, for example, limits NF-κB [66–68], reduces IL-6, NOS-2 and PGE2 synthase in bone marrow-derived macrophages [69,70] and is reported to block LPS binding to CD44 [71]. Furthermore, high molecular weight HA reduces inflammation upon binding to CD44 [72]. However, these effects are equally likely to increase the macrophage glycocalyx, which was not tested in these prior studies. High heparanase activity is observed in COVID-19 and is associated with increased disease severity and pro-inflammatory IL-6, TNF, IL-1 and CCL2 production [73]. These effects are reversed by heparanase blockade [74] though the impact on glycocalyx is untested. In addition, deletion of SDC-1 (covalently linked to HS and CS [75]) enhances inflammation in an asthma model [53]. Whether these manipulations restore negative regulation and/or activating receptor shielding is unknown. However, our data provide evidence that macrophages may sense health, and when to inflame, through their glycocalyx.

In summary, macrophages express genes involved in glycocalyx biosynthesis, which suggests components are not simply adsorbed from the local environment. Differential regulation of glycocalyx density on macrophages in environmentally exposed sites makes sense, as does a degradation of the glycocalyx to signal a switch away from a healthy environment to inflammation. Regaining glycocalyx on macrophages is likely important to switch on repair programmes and also prevent the development of autoimmunity upon efferocytosis of apoptotic cells and the removal of host cell debris. Manipulation of glycocalyx components, therefore, provides a strategy to increase or decrease the onset of inflammation.

## Methods

### Animals

Male and female C57BL/6 mice (10–12 weeks old) were housed in the Biological Services Facility at 22 °C to 24 °C, with 45% to 55% humidity and a 12:12-h light-dark schedule. All procedures were approved by the University of Manchester Animal Welfare and Ethical Review Body and the Home Office UK in accordance with the UK Animals (Scientific Procedures) Act, 1986. Ten male B6.SJL-Ptprca Pepcb/BoyJ mice (CD45.1^+^ CD45.2^-^) were provided by Dr Grace Mallett and Joshua Briggs at the University of Manchester. All C57BL/6 mice (CD45.1^-^ CD45.2^+^) were purchased from ENVIGO UK Ltd. For the immunofluorescence study, one lobe of lungs from naïve wildtype female mice was directly collected in formalin after euthanising the mice with pentobarbitone overdose. The other four lobes of the lungs were digested into single cell suspension for flow cytometry studies.

### Isolation of mouse bronchoalveolar lavage macrophages

After the mice underwent terminal anaesthesia with pentobarbital overdose. The trachea was catheterised, and alveolar cells were collected in the bronchoalveolar lavage (BAL) by washing the lung five times, each with 1.5 ml 1:1000 EDTA (Thermo Fisher Scientific, AM9260G) in PBS, using a 26 G needle and syringe. The cell-containing PBS was centrifuged at 500 xg for 5 min, and was mixed with red blood cell lysing buffer (Sigma-Aldrich, R7757) and incubated at room temperature for 3 min. The cells were washed with PBS containing 2% FBS and 2 mM EDTA, and resuspended in PBS.

### Isolation and digestion of mice lungs

After the male and female mice were euthanised with pentobarbitone overdose, the lung was collected in formalin for immunofluorescence staining, or HBSS (Sigma-Aldrich, H9269) for flow cytometry analysis. The lungs were homogenised and digested in Liberase TM (100 ug/ml) (Millipore Sigma, 5401,127,001) and DNase I (50 ug/ml) (Sigma, 11,284,932,001), on a shaker, at 37 °C for 30 min. After the incubation, the digestion was stopped by adding the same volume of PBS containing 2% FBS and 2 mM EDTA (Thermo Fisher Scientific, AM9260G). The tissue was then passed through a 70-µm sieve, which was washed with HBSS to obtain the cells. The cells were washed and resuspended. The cells were mixed with red blood cell lysing buffer (Sigma-Aldrich, R7757) and incubated at room temperature for 3 min, followed by another wash. After resuspending the cells in 1 ml PBS, 10 µl of cell-containing PBS was mixed with 1 µl methyl orange and 89 µl PBS in a fresh tube for cell counting. The count was conducted by automated cell counters & analysers (ChemomeTec, NucleoCounter NC-250) and the ChemoMetec Nucleo NC-250 software (ChemomeTec). After the cell count, the cells were passed to the staining step for flow cytometry analysis.

### Influenza infection

Balanced male and female mice were anaesthetised with isoflurane and intranasally infected with 30 µL containing 5 plaque-forming units (PFU) of influenza A virus, Puerto Rico/8/34 (PR8), H1N1 or the same volume of PBS as a control. Mouse weight was measured daily and euthanised at day 7 or day 14 by intraperitoneal injection of 5 mg pentobarbitone. Bronchoalveolar lavage was conducted by instilling and recovering 5 ml of Hanks’ Balanced Salt solution (HBSS), with 0.5 mM EDTA (MERCK, 324,506), from the lungs via an intratracheal cannula. The remaining lung tissue was resected. The left lobes (superior and inferior) were fixed in formalin and processed for paraffin wax embedding. The remaining right (superior, middle and inferior) lobes were homogenised and digested in 1 ml HBSS with Liberase TM (100 µg/ml; Millipore Sigma, 5401,127,001) and DNase I (50 ug/ml; Sigma, 11,284,932,001) in HBSS, on a shaker, at 37 °C for 30 min. The digestion was stopped with 4 ml of HBSS containing 2% FBS and 2 mM EDTA (Thermo Fisher Scientific, AM9260G). The tissue was passed through a 70 µm sieve, washed with HBSS, and collected by centrifugation at 500 g for 5 min. Red blood cells were removed by incubating with 3 ml of lysis buffer (Sigma-Aldrich, R7757) for three minutes at RT. After washing, cell viability was assessed using methyl orange and viability was enumerated on an automated cell counter (ChemomeTec, Nucleo-Counter NC-250) and the ChemoMetec Nucleo NC-250 software (ChemomeTec).

### CD45.1+ BMDMs intranasally transfer model

For the cell transfer experiment, bone marrow cells from male B6. SJL-Ptprca Pepcb/BoyJ mice (CD45.1^-^ CD45.2^+^) were collected and derived with M-CSF for 7 days into macrophages. BMDMs derived from 3, or 4 mice were pooled together, and intranasally given to 8, or 10 female wildtype C57BL/6 mice (CD45.1^-^ CD45.2^+^), respectively. Each wildtype C57BL/6 mouse was instilled intranasally with 5 × 10^5^ of the M-CSF-derived BMDMs in 25 ml PBS. The BAL fluid and lungs of the CD45.1^-^ CD45.2^+^ wildtype C57BL/6 mice were collected 7 days or 14 days after the installation. The lungs were enzymatically digested into a single-cell suspension. Cells from the BAL fluid and the digested lungs were stained for flow cytometry analysis.

### Poly I:C treatment of BAL alveolar macrophages

Female wildtype C57BL/6 mice were euthanised with pentobarbitone overdose. Cells collected from bronchoalveolar lavage fluid were counted and seeded into 12-well culture plates (Thermo Fisher Scientific, 150,628), at 1 × 10^5^ per well. The cells were cultured in high FBS culture media: RPMI 1640 Medium (Thermo Fisher Scientific, 11,879,020) with 20% FBS (Gibco, 11,533,387), 100 U/ml penicillin (Gibco, 15,140,122), and 100 ug/ml streptomycin (Gibco, 15,140,122), and 20 mM HEPES solution (Sigma-Aldrich, H0887); or low FBS culture media: 20% FBS replaced with 0.5% FBS. Polylysine-polycytidylic acid (Poly I:C) (Invivogen, 31,852-29–6) at 10 ug/ml were applied to stimulate the cells. The control group were given the equivalent volume of culture media. The cells were incubated in an incubator at 5% CO_2_, 37°C. After 24 h of incubation, cell-free culture supernatants were collected. The cells were washed with Dulbecco’s Phosphate Buffered Salin (PBS) (Sigma-Aldrich, RNBK4357) to remove unattached cells. PBS were added, followed by scrubbing and washing to collect macrophages attaching to the wells. Collected cells were immediately stained with antibodies for a flow cytometry study.

### Studies using bone marrow-derived cells

For *in vitro* studies, bone marrow cells, collected from female wildtype C57BL/6 mice, were cultured with 20 ng/ml recombinant murine M-CSF (PeproTech, 315–02) or 20 ng/ml recombinant murine GM-CSF (PeproTech, 315–03), in RPMI 1640 Medium mixture mentioned above. On day 4, media was refreshed with 20 ng/ml M-CSF or 20 ng/m GM-CSF and on day 8, non-adherent cells were removed. Adherent cells were recovered by scraping and centrifugation. In some experiments, the cells were harvested at day 3 or day 14. Cells harvested at day 14 had half of the medium refreshed on day 7 and day 10. In some experiments, BMDMs were further polarised for 24 h with the RPMI 1640 Medium mixture with 20 ng/ml recombinant murine IFN-γ (PeproTech, 315–05), 10 ng/ml Lipopolysaccharide (Invivogen, tlrl-eblps); or 20 ng/ml recombinant murine IL-4 (PeproTech, 214–14), before scraping. Conditions based on previous studies [76]. In all polarisations, cells were subjected to flow cytometric analysis. In some experiments where the RNA transcriptome was analysed, polarised adherent cells were incubated in 350 µl of RLT buffer (Qiagen, 74,004) mixed with 1:100 beta-mercaptoethanol (Sigma-Aldrich, 60–24–2) to produce cell lysis and stored at −80 °C.

### Flow cytometry staining and analysis

Approximately 2 × 10^6^ cells were incubated with mouse FcR Blocking Reagent (1:500, Miltenyl Biotec, Germany, 5210,502,523) and zombie UV (1:1000, BioLegend, USA, 423,107) in 50 µl PBS for 15 min at RT. After 50 µl of 2% paraformaldehyde fixation at RT for 10 min, cells were stained with the indicated directly conjugated or unconjugated (followed by a secondary detection antibody) (table S1) antibodies in 50 µl PBS with 1% FBS, overnight at 4 °C. After washing and fixation with 50 µl 2% paraformaldehyde. For intracellular staining, the cells were fixed and permeabilised with eBioscience™ Foxp3 / Transcription Factor Staining Buffer Set (Invitrogen, 00–5523–00) according to the manufacturer’s instructions. The cells were stained with an intracellular antibody for 30 min, then fixed with 50 µl 2% paraformaldehyde. Samples were run on a BD FAC Fortessa Flow Cytometer (BD Bioscience, UK), using the FACS Diva software (BD Bioscience, Belgium) and analysed with the FlowJo software (Tree Star, USA).

### Confocal microscopy

The formalin-fixed and paraffin-embedded lung tissues were trimmed and attached to glass slides (MERCK, CLS294875 × 25). The slides were deparaffinized with xylene and ethanol. Antigen retrieval was accomplished at 95 °C for 20 min in 400 ml Tris-EDTA buffer (Sigma-Aldrich, T9285). Slides were then permeabilised with 200 ml 0.5% Triton X-100 (Sigma-Aldrich, X100) and blocking buffer applied (1% FBS, 2% donkey serum (MERCK, D9663) and 0.05% Tween-20 (MERCK. P2287)) to cover the tissue on the glass slides for 30 min. Primary antibodies were applied to cover the tissue overnight at 4 °C, and if required, followed by secondary antibodies at RT for 1 hour (tables S2). The slides were stained with equivalent amount of 0.2 μg/ml 4′,6-diamidino-2-phenylindole (DAPI, Thermo Scientific, 62,248) in H_2_O for 5 min. The slides were washed with 200 ml 0.1% Tween 20 (Sigma-Aldrich, 11,332,465,001) in PBS and two washes with 200 ml PBS after each step. The slides were mounted with ProLong™ Gold Antifade Mountant (Invitrogen, P36934), and covered with cover slides (Corning, CLS2975224). Slides were imaged using an EVOS FL Auto imaging system (Thermo Fisher Scientific) and analysed using Image-J Software.

### HILIC-MS/MS glycosaminoglycan disaccharide analysis

GAG disaccharide analysis was performed via HILIC-MS/MS as described elsewhere (https://dx.doi.org/10.17504/protocols.io.x54v956n1l3e/v1) [77]. Briefly, GAGs were liberated from other biomolecules via pronase and Dnase I digestion. Liberated GAGs were enriched by centrifugation through a molecular weight cut off filter and were depolymerised with either a mix of Heparinase I, II, III or Chondroitinase ABC (to yield HS and CS/DS/HA disaccharides, respectively). Disaccharides were passed over a solid phase extraction plate, lyophilised, reconstituted in water and diluted into 75% acetonitrile, 44 mM ammonium acetate for HILIC-MS/MS analysis. HILIC-MS/MS was performed on a Shimadzu Nexera LC 30 CE coupled to a SCIEX 7600 ZenoTOF mass spectrometer with an InfinityLab Poroshell 120 HILIC-Z column and guard.

### RNA isolation, reverse transcription, and rt-qpcr

RNA was isolated using the RNeasy Micro Kit (Qiagen, 74,004) according to the manufacturer’s instructions. The total RNA concentration was quantified with a NanoDrop 2000/2000c Spectrophotometer (Thermo Fisher Scientific, ND-2000). Reverse transcription was conducted using the High-Capacity RNA-to-cDNA Kit (Applied Biosystems, 4387,406) and 100 ng RNA was used in each reverse transcription. The samples were incubated at 37 °C for 60 min, then at 95 °C for 5 min for heat inactivation and stored at 4 °C. After reverse transcription, the cDNA was diluted 1:4 in nuclease-free water. Reactions were performed in triplicate on a MicroAmp™ Optical 384-Well Reaction Plate with Barcode (Applied Biosystems, 4309,849). Each qPCR reaction contained 5 μl PowerUp SYBR Green Master Mix (Applied Biosystems, A25742), 0.6 μl 10 μM each of forward primer and reverse primer, 2.4 μl ddH2O and 2 μl cDNA to perform a 10-μl reaction volume. was analysed on a 7900HT Fast Real-Time PCR System (Applied Biosystems). The PCR cycle included 95 °C for 5 min, 40 × 15 s at 95 °C, 20 s at 57 °C and 20 s at 72 °C, followed by 95 °C, 60 °C and 95 °C for 15 s each, sequentially. Relative mRNA levels were calculated compared to the average mRNA expression of the housekeeping gene beta-2-microglobulin (B2m). Relative mRNA expression and fold changes were calculated based on the ΔΔCT method. Ct, ΔCt, ΔΔCt and 2-ΔΔCt were calculated (Livak & Schmittgen, 2001). Primers: KiCqStart™ Primers purchased from MERCK: *b2m* (M_B2m_1), *nos-2* (M_Nos2_1), *chi3l-1* (M_Chi3l1_1), *b4galt-7* (M_B4galt7_1), *extl-1* (M_Extl1_1), *extl-2* (M_Extl2_1), *extl-3* (M_Extl3_1), *sdc-1* (M_Sdc1_1), *sdc-2* (M_Sdc2_1), *sdc-3* (M_Sdc3_3), *sdc-4* (M_Sdc4_1), *has-1* (M_Has1_1), *has-2* (M_Has2_1), *has-3* (M_Has3_1), *mmp-9* (M_Mmp9_1), *mmp-12* (M_Mmp12_1), and *mmp-13* (M_Mmp13_1).

### Statistical analysis

All statistics were calculated using GraphPad Prism 10.2.0 (Graph-Pad Software Inc., La Jolla, *CA*). Analysis was carried out on sample size. When comparing between two groups, an unpaired *t-test* was performed to determine statistical significance. When comparing among three or more groups, an analysis of variance (ANOVA) was performed. Error bars indicate SEM.

## Supplementary Material

Supplementary material associated with this article can be found, in the online version, at doi:10.1016/j.matbio.2026.102033.

Supplementary Information

## Data Availability

Data will be made available on request.
