# Supplementary material for: Airway macrophage specific glycocalyx expression and remodeling following viral infection
Source: Matrix Biol. Author manuscript; Available in PMC 2026 Aug 13. (PMC7619357; doi:10.1016/j.matbio.2026.102033)
Supplement: Supplementary file 1 [file EMS216254-supplement-Supplementary_Information.docx]

**Supplementary Information**

**Table S1: Reagents and antibodies for flow cytometry staining.** *MERCK, ASMBIO, * MBL International, *Novus Biologicals, *Proteintech, *R&D System, *Amsbio, *Invitrogen, *Bioworld, *Vector Biology

| **Reagent** | **Clone** | **Host/Isotype** | **Dilution** | **Conjugate** | **Catalogue** |
| --- | --- | --- | --- | --- | --- |
| *Wheat germ agglutinin (WGA) |  |  | 1:1000 | FITC | L4895 |
| Anti-mouse CD45 | 30-F11 | Rat IgG2b, κ | 1:200 | Brilliant Violet 510 | 103137 |
| Anti-mouse Ly-6G | 1A8 | Rat IgG2a, κ | 1:200 | APC/Cyanine7 | 127624 |
| Anti-mouse/ human CD11b | M1/70 | Rat IgG2b, κ | 1:500 | Brilliant Violet 711 | 101242 |
| Anti-mouse CD11c | N418 | Armenian Hamster IgG | 1:400 | Brilliant Violet 605 | 117334 |
| Anti-mouse CD64 (FcγRI) | X54-5/7.1 | Mouse IgG1, κ | 1:200 | Brilliant Violet 421 | 139309 |
| Ab Heparan Sulfate | F58-10E4 | Mouse IgM, κ | 1:200 | Biotin | 370255-B-50-L |
| Anti-mouse Ly-6C | HK1.4 | Rat IgG2c, κ | 1:200 | Alexa Fluor 700 | 128023 |
| Anti-mouse CD138 | 281-2 | Rat IgG2a, κ | 1:200 | PE/Cyanine 7 | 142513 |
| Anti-mouse CD170 (Siglec-F) | S17007L | Rat IgG2a, κ | 1:200 | PE/Dazzle™ 594 | 155529 |
| Anti-mouse CD64 (FcγRI) | X54-5/7.1 | Mouse IgG1, κ | 1:200 | PE/Cyanine 7 | 139313 |
| Anti-mouse CD284 (TLR4) | SA15-21 | Rat IgG2a, κ | 1:200 | PE | 145403 |
| Anti-mouse /human CD44 | IM7 | Rat IgG2b, κ | 1:200 | PE/Cyanine 5 | 103009 |
| Anti-mouse CD45.1 | A20 | Mouse (A.SW) IgG2a, κ | 1:200 | Brilliant Violet 650 | 110736 |
| Anti-mouse CD45.2 | 104 | Mouse (SJL) IgG2a, κ | 1:200 | Brilliant Violet 785 | 109839 |
| Hyaluronan Binding Protein |  | Recombinant human versican G1 domain(rHABP) expressd in *E.coli* | 1:200 | Biotin | AMS.HKD-BC41 |
| Anti-Syndecan 4 antibody | 112 | Rabbit IgG | 1:200 | PE | ab279590 |
| anti-mouse F4/80 Antibody | BM8 | Rat IgG2a, κ | 1:200 | Brilliant Violet 605 | 123133 |
| Endorepellin/Perlecan/Heparan Sulfate Proteoglycan Antibody | A7L6 | Rat IgG2a, κ | 1:1000 | FITC | NBP2-47695F |
| Maackia amurensis Lectin (MAA/MAL I) |  | Amur maackia seeds | 1:1000 | FITC | 21761036-1 |
| Lycopersicon Esculentum (Tomato) Lectin (LEL, TL) |  | Tomato | 1:1000 | DyLight 649 | L32472 |
| Sambucus Nigra Lectin (SNA, EBL) |  | Elderberry bark | 1:1000 | Cy5 | CL-1305-1 |
| HPSE Polyclonal antibody |  | Polyclonal Rabbit IgG | 1:200 |  | 24529-1 |
| Streptavidin |  |  | 1:200 | Alexa Fluor 594 | 405240 |
| Streptavidin |  |  | 1:200 | Alexa Fluor 647 | 405237 |
| Rabbit IgG NorthernLights™ NL557-conjugated Antibody |  | Polyclonal Donkey IgG | 1:200 | NorthernLights 557 | NL004 |

**Table S2: Reagents and dilutions for immunofluorescence staining**

| **Reagent** | **Clone** | **Host/Isotype** | **Dilution** | **Conjugate** | **Catalogue** |
| --- | --- | --- | --- | --- | --- |
| Wheat germ agglutinin (WGA) |  |  | 1:2000 | FITC | MERCK L4895 |
| CD64 Rabbit | 008 | Rabbit / IgG | 1:200 | Unconjugated | Invitrogen MA5-29705 |
| Rat Anti-Mouse Siglec-F | E50-2440 | Rat (LOU) IgG2a, κ | 1:200 | Unconjugated | 552125 BD Pharmingen |
| Rabbit IgG NorthernLights™ NL637 |  | Polyclonal Donkey IgG | 1:200 | NorthernLights 637 | R&D System NL005 |
| Donkey anti-Rat IgG (H+L) Highly |  | Polyclonal Donkey IgG | 1:200 | Alexa Fluor 594 | Invitrogen A21209 |

**Supplementary Figure 1: Gating strategy for lung interstitial macrophages (IMs) and alveolar macrophages (AMs).** Cells were isolated from the digested lungs of female C57BL/6 mice. After staining, the cells were run through a BD Fortessa flow cytometer (**A**) and singlets were identified (**B**). Live cells (**C**) and CD45^+^ cells (**D**) were gated. Cells positive for Ly6G, CD3, or CD19 were gated out to remove T cells and B cells (**E**). Cells either CD11c or CD11b positive were gated (**F**), followed by CD64^+^ cells to identify macrophages (**G**). The remaining cells with CD11b^high^ were classified as IMs, and those with CD11c^high^ were classified as AMs (**H**).

**Supplementary Figure 2: Flow cytometry comparison of the glycocalyx on BM progenitor cells and unpolarized BMDMs.** The femurs of C57BL/6 female mice were flushed with HBSS to collect bone marrow cells. Bone marrow progenitor cells, cultured with 20 ng/ml M-CSF for 3 days were compared with BMDMs that were derived with M-CSF for 7 days and 14 days, on general glycocalyx (WGA lectin, detects the N-acetyl-glucosamine) (**A**), SDC-1 (**B**), CD44 (**C**) and HS (**D**). The medium fluorescence intensity (MFI) and percentage of cells positive are shown. Each dot represents a biological repeat (cells derived from a different individual mouse), data are pooled from four independent experiments (*n* = 14) and presented as the mean ± SEM. Data was analysed with one-way ANOVA. BMDMs cultured in 20 ng/ml M-CSF or GM-CSF for 7 days to derive the unpolarized (M0) M-CSF-derived BMDMs and GM-CSF-derived BMDMs. The BMDMs derived with the two cytokines were compared on general glycocalyx (**E**), SDC-1 (**F**), CD44 (**G**). Representative histograms are shown: M-CSF-derived BMDMs (red), GM-CSF-derived BMDMs (blue), and fluorescence minus one (FMO) control (grey). Each dot represents a biological repeat (cells derived from a different individual mouse), data are pooled from four independent experiments (*n* = 15) and presented as the mean ± SEM. Data was analysed with unpaired t-tests. No significant differences were found.

**Supplementary Figure 3: GM-CSF and M-CSF-derived BMDMs express differential levels of genes that regulate glycocalyx synthesis and degradation**. Bone marrow cells were derived with M-CSF or GM-CSF. The glycocalyx-relative mRNA expression was measured in GM-CSF- and M-CSF-differentiated BMDMs. The qPCR data are normalised to the housekeeping gene *b2m* and displayed as the relative expression levels in comparison to the expression level of *b2m*. *Has-1* (**A**), *has-2* (**B**), *has-3* (**C**), *sdc-1* (**D**), *sdc-2* (**E**), *sdc-3* (**F**), *sdc-4* (**G**), *extl-1* (**H**), *extl-2* (**I**), *extl-3* (**J**), *b4galt7* (**K**), *mmp-9* (**L**), *mmp-12* (**M**), and *mmp-13* (**N**) Each dot represents a biological repeat (cells derived from a different individual mouse), data are pooled from three independent experiments (*n* = 10), shown as the mean ± SEM. Data was analysed with unpaired t-tests. *, P ≤ 0.05.

**Supplementary Figure 4**: **Expression of M1 and M2 polarisation markers in BMDMs.** The mRNA expression of *nos-2* (**A**) and *chi3l-1* (**B**), display as fold change over the level of unpolarised M0, in M-CSF-derived BMDMs. Data are representative of two independent experiments. The mRNA expression of *nos-2* (**C**) and *chi3l-1* (**D**), display as fold change over the level of unpolarised M0, in GM-CSF-derived BMDMs. Each dot represents a biological repeat (cells derived from a different individual mouse), data are pooled from three independent experiments and plotted as the mean ± SEM (M-CSF, *n* = 11*;* GM-CSF, *n* = 14). Data was analysed with unpaired t-tests. **, P ≤ 0.01; ***, P ≤ 0.001.

**Supplementary Figure 5**: **Relative expression levels of glycocalyx-related genes in M-CSF- and GM-CSF-derived BMDMs in M1 and M2 polarising stimuli.** M-CSF-derived BMDMs and GM-CSF-derived BMDMs were left unstimulated (green) or polarized to classically (M1) (red) and alternatively activated (M2) (blue) macrophages. The relative mRNA expression levels were measured according to the housekeeping gene *b2m*. *Has-1* (**A**), *has-2* (**B**), *has-3* (**C**), *sdc-1* (**D**), *sdc-2* (**E**), *sdc-3* (**F**), *sdc-4* (**G**), *extl-1* (**H**), *extl-2* (**I**), *extl-3* (**J**), *b4galt7* (**K**), *mmp-9* (**L**), *mmp-12* (**M**), and *mmp-13* (**N**). Each dot represents a biological repeat (cells derived from a different individual mouse), data are pooled from three independent experiments and plotted as the mean ± SEM. M=CSF, *n* = 11; GM-CSF, *n* = 14. Data was analysed with unpaired t-tests. *, P ≤ 0.05; **, P ≤ 0.01; ***, P ≤ 0.001; ****, P ≤ 0.0001.

**Supplementary Figure 6: SDC-1 levels remain unchanged following the flu infection on monocytes, eosinophils and neutrophils**. At day 0, mice were intranasally given 5 PFU IAV PR8 in 25 ul PBS, or given the same volume of PBS (25 µl) as control. On day 7, the lung tissue from both groups was harvested. Myeloid cells were distinguished by a flow cytometer, including neutrophils (CD45^+^ Ly6G^high^ CD11b^+^), airway macrophages (CD45^+^ Ly6G^medium/low^ CD64^+^ Siglec F^+^), eosinophils (CD45^+^ Ly6G^medium/low^ CD64^-^ Siglec F^+^), IMs (CD45^+^ Ly6G^medium/low^ CD64^+^ Siglec F^-^ CD11b^+^ CD11c^+^), and monocytes (CD45^+^ Ly6G^medium/low^ CD64^+^ Siglec F^-^ CD11b^+^ CD11c^-^) (**A**).The MFI, and percentage, of general glycocalyx showed by WGA lectin staining of monocytes (**B**), eosinophils (**C**), and neutrophils (**D**). The MFI and percentage of monocytes (**E**), eosinophils (**F**), and neutrophils (**G**) positive for SDC-1 expression. The histograms are representative of one mouse per group. Each dot represents a biological repeat (cells derived from a different individual mouse) (Naïve, *n* = 14; Infected, *n* = 14), data are pooled from three independent experiments data and plotted as mean ± SEM and was analysed by unpaired t-test. *, P ≤ 0.05; **, P ≤ 0.01; ***, P ≤ 0.001; ****, P ≤ 0.0001.
